# The crystal structures and Hirshfeld surface analysis of the 2-iodophenyl- and 4,5-difluoro-2-iodophenyl derivatives of benzenesulfonamide

**DOI:** 10.1107/S2056989025006656

**Published:** 2025-08-07

**Authors:** Sundarasamy Madhan, MohamedHanifa NizamMohideen, Vinayagam Pavunkumar, Arasambattu K. MohanaKrishnan

**Affiliations:** a Department of Physics The New College Chennai 600 014 University of Madras,Tamil Nadu India; b Department of Organic Chemistry University of Madras, Guindy Campus Chennai-600 025 Tamilnadu India; Vienna University of Technology, Austria

**Keywords:** crystal structure, 2-iodo­phen­yl, 4,5-di­fluoro, benzene­sulfonamide, π–π inter­actions, hydrogen bonding, Hirshfeld surface analysis

## Abstract

Two new benzene­sulfonyl derivatives, *N*-(2-iodo­phen­yl)benzene­sulfonamide, C_12_H_10_INO_2_, (**I**), and *N*-(4,5-di­fluoro-2-iodo­phen­yl)benzene­sulfonamide, C_12_H_8_F_2_INO_2_S, (**II**) were synthesized and structurally characterized. In both mol­ecular structures, the conformation of the N—C bond in the –SO_2_—NH—C segment is *gauche* relative to the S=O bond. For (**I**), the crystal packing is dominated by N—H⋯O hydrogen-bonding inter­actions that link the mol­ecules into chains extending parallel to [010]. In the case of (**II**), the mol­ecules are linked by N—H⋯O(S) hydrogen bonds into dimers that are located on centers of inversion. These findings are consistent with the results of Hirshfeld surface analyses.

## Abstract 2

*N*-(2-Iodo­phen­yl)benzene­sulfonamide, C_12_H_10_INO_2_, and *N*-(4,5-di­fluoro-2-iodo­phen­yl)benzene­sulfonamide, C_12_H_8_F_2_INO_2_S differ only in the replacement of two H atoms by F atoms, which changes the symmetry from *P*2_1_/*c* to *P*1 and is accompanied by different mol­ecular conformations and packing features.

## 1. Chemical context

Sulfonamide-containing compounds, often referred to as sulfa drugs, form a significant class of pharmacologically active agents. These mol­ecules, which may incorporate one or more pharmacological scaffolds, demonstrate a broad spectrum of biological activities including anti­viral, anti­cancer, anti­bacterial, anti-carbonic anhydrase (CA), diuretic, COX-2 inhibitory, and protease inhibitory effects (Madhan *et al.*, 2024*a* ▸,*b* ▸,*c* ▸). The sulfonamide moiety is recognized as an important structural unit in medicinal chemistry and is present in many widely marketed drugs (Supuran, 2003 ▸; Elgemeie *et al.*, 2019 ▸). Since their discovery, sulfonamides have been extensively used as anti­biotics (Zhao *et al.*, 2016 ▸), particularly for treating infections like malaria, tuberculosis, or HIV, by targeting the di­hydro­pteroate synthase (DHPS) pathway (Dennis *et al.*, 2018 ▸). Even after the advent of penicillin, sulfa drugs have retained their relevance in clinical settings due to their diverse therapeutic actions, including anti­tumor, anti­cancer, and anti­thyroid activities (Scozzafava *et al.*, 2003 ▸). Various sulfonamide derivatives serve as chemotherapeutic agents, exhibiting anti­bacterial, anti­fungal, anti­tumor, and hypoglycemic properties (Chohan *et al.*, 2010 ▸; El-Sayed *et al.*, 2011 ▸ Seri *et al.*, 2000 ▸). Benzene­sulfonamide derivatives are particularly known for their anti­tumor and anti­fungal activities. Crystallographic studies of these compounds reveal structural parameters consistent with other sulfonamide-based mol­ecules (Chakkaravarthi *et al.*, 2007 ▸; Li & Yang, 2006 ▸). Continued inter­est in sulfonamides stems from their enduring role in treating bacterial infections, their chemical versatility, and their effectiveness despite the rise of newer anti­biotic classes. Modern synthetic approaches aim to produce sulfon­amide-functionalized heterocycles with enhanced anti­viral and anti­microbial profiles (Madhan *et al.*, 2024*a* ▸). Research into N-sulfonyl­ated I and F atom-substituted compounds is motivated by the observed enhancement of biological activity. Hence, the introduction of fluorine atoms into drugs is increasingly common due to the strong electron-withdrawing character and small atomic radius of the fluorine atom, which significantly influences the physiological, pharmacological and metabolic properties of a compound (Mueller *et al.*, 2007 ▸; Purser *et al.*, 2008 ▸). The availability of multiple aromatic groups in N-sulfonyl­ated 2-iodo­phenyl imposes also the possibility for versatile stacking patterns, which may be competitive to the conventional hydrogen-bonding inter­actions in the crystal packing.
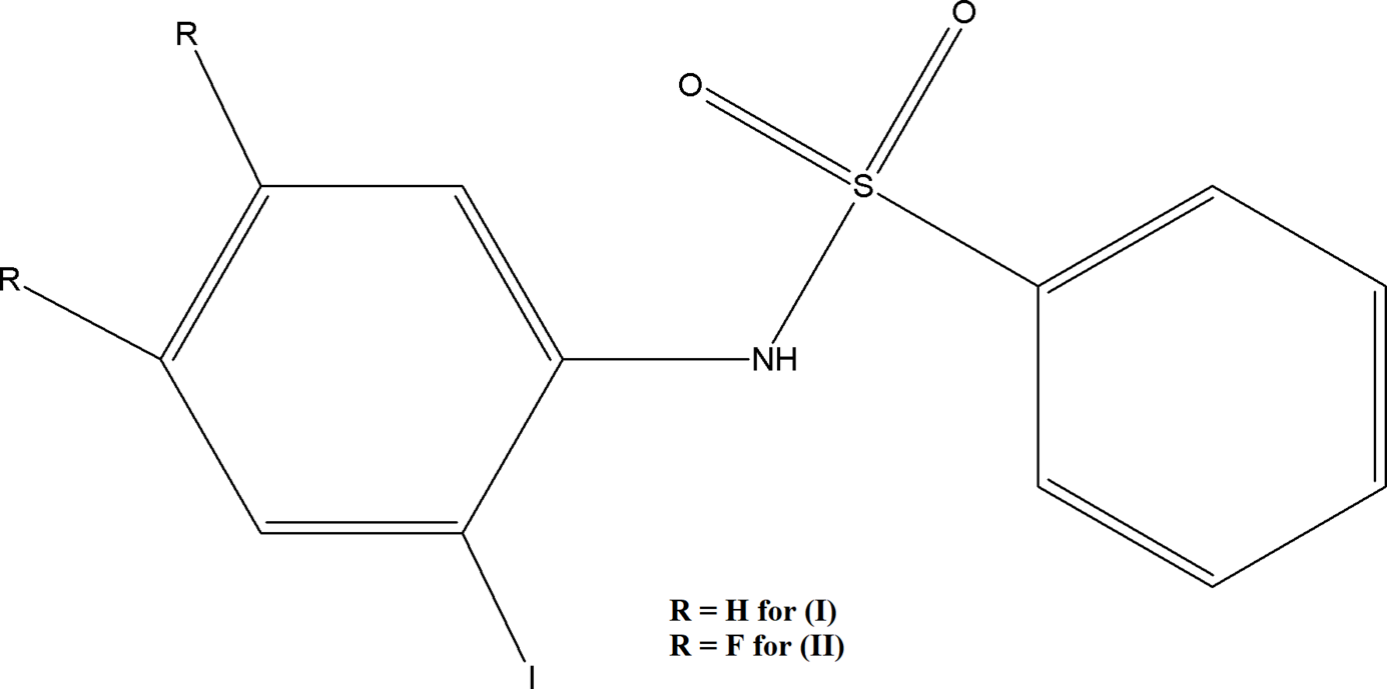


In the context given above, we report herein the crystal structure determinations and Hirshfeld surface analyses of two new 2-iodo­phenyl benzene­sulfonamides: *N*-(2-iodo­phen­yl)benzene­sulfonamide, C_12_H_10_INO_2_, (**I**), and *N*-(4,5-di­fluoro-2-iodo­phen­yl)benzene­sulfonamide, C_12_H_8_F_2_INO_2_S, (**II**), which feature a complex inter­play of weak hydrogen bonding and π–π inter­actions.

## 2. Structural commentary

**Figure 1:**
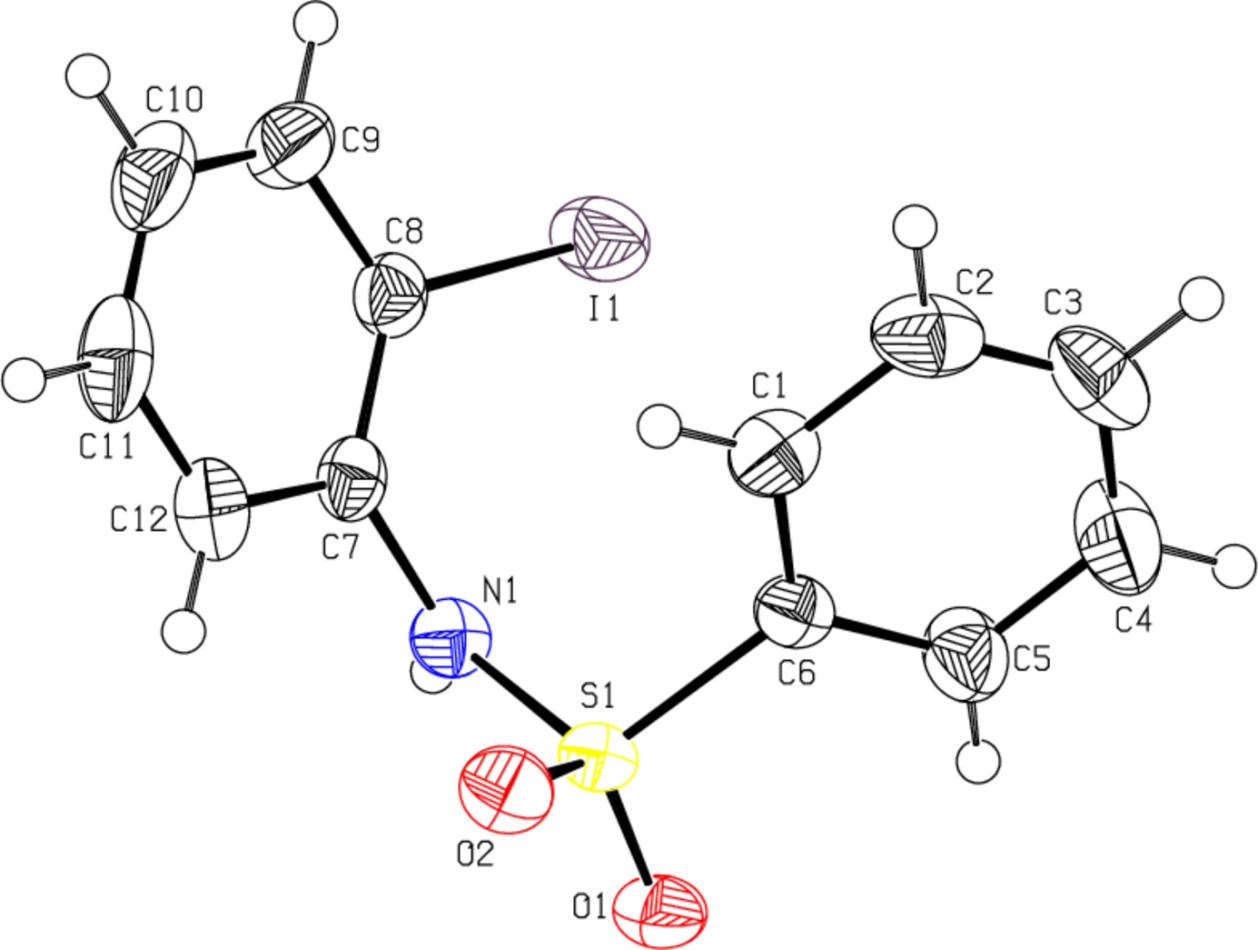
The mol­ecular structure of compound (**I**), with atom labeling and displacement ellipsoids drawn at the 50% probability level.

The mol­ecular structures of (**I**) and (**II**) are shown in Figs. 1 ▸ and 2 ▸, respectively. In both cases, the conformation of the N—C bond in the –SO_2_—NH—C segment is *gauche* relative to the S=O bond. The mol­ecule is twisted at the S—N bond with a torsion angle of C7—N1—S1—C6 = −69.0 (2)° for (**I**) and −61.1 (6)° for (**II**) compared to the values of −72.83 (15) and 61.9 (3)° in *N*-(phen­yl)-2-nitro­benzene­sulfonamide (Chaithanya *et al.*, 2012*a* ▸) and 4-nitro-*N*-phenyl­benzene­sulfonamide (Chaithanya *et al.*, 2012*b* ▸), respectively. The two benzene rings are tilted relative to each other by 44.1 (1)° for (**I**) and 73.1 (1)° for (**II**). The mol­ecular configuration of (**II**) is stabilized by a weak intra­molecular hydrogen bond C12—H12⋯O1 (Table 1 ▸) with one of the sulfone O-atoms as acceptor, which generates an *S*(6) ring motif. Other structural parameters (bond lengths and angles) in the mol­ecules of (**I**) and (**II**) agree well with those reported for related compounds (Madhan *et al.*, 2022 ▸, 2023*a* ▸,*b* ▸, 2024*a* ▸,*b* ▸,*c* ▸).

## 3. Supra­molecular features

**Figure 3:**
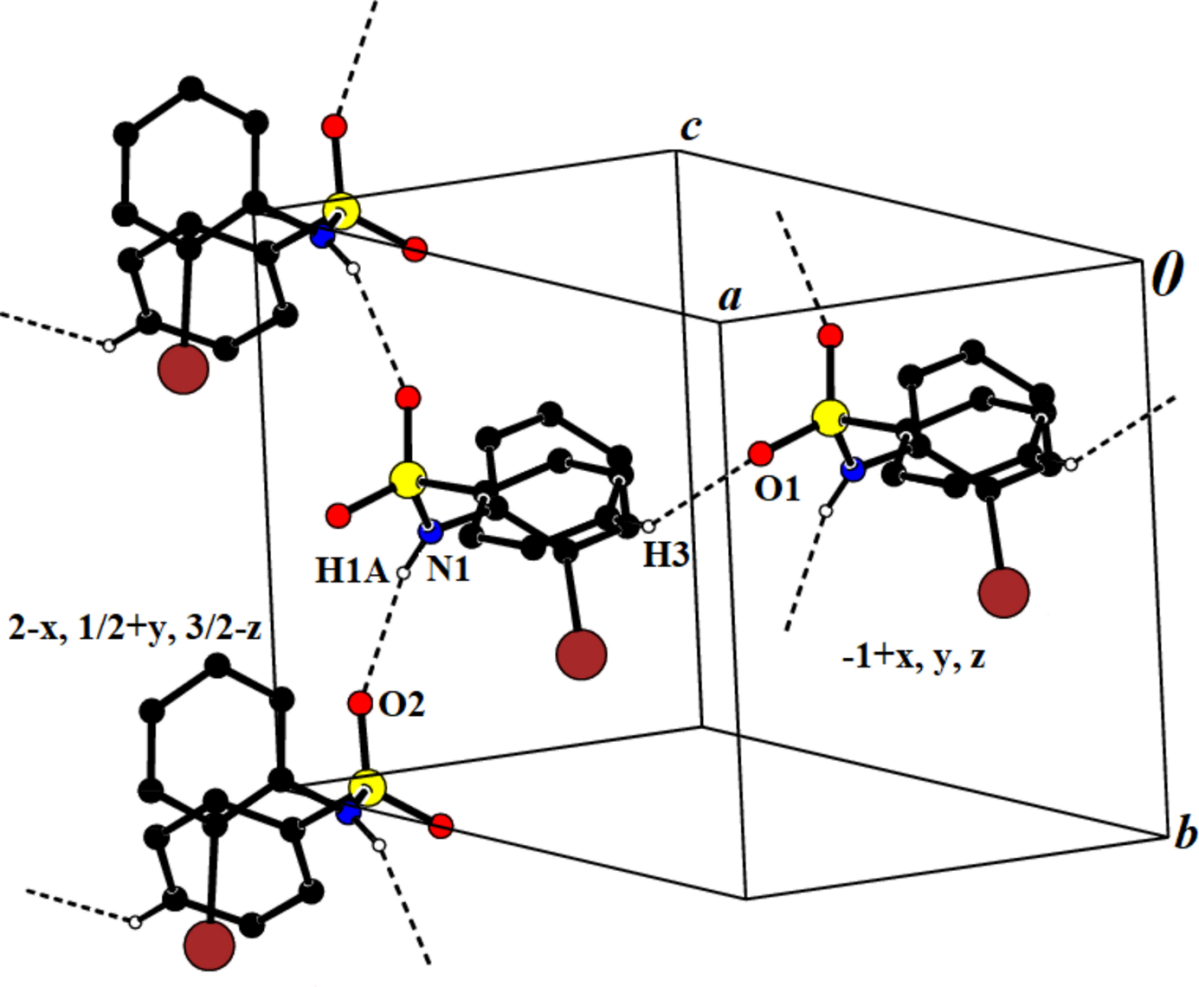
Crystal packing of compound (**I**), showing the N—H⋯O and C—H⋯O inter­action that link the mol­ecules into chains.

In the crystal structure of (**I**), inter­molecular N—H⋯O hydrogen-bonding inter­actions (Table 1 ▸) link the mol­ecules into *C*(4) chains (Etter *et al.*, 1990 ▸) running parallel to [010] while C—H⋯O inter­actions inter­link these chains (Fig. 3 ▸). In addition, π–π inter­actions are present with a centroid-to-centroid distance *Cg*2⋯*Cg*2 (2 − *x*, 1 − *y*, 1 − *z*) = 3.747 (2) Å and a slippage of 1.035 Å (*Cg*2 is the centroid of phenyl ring C7–C1).

**Figure 4:**
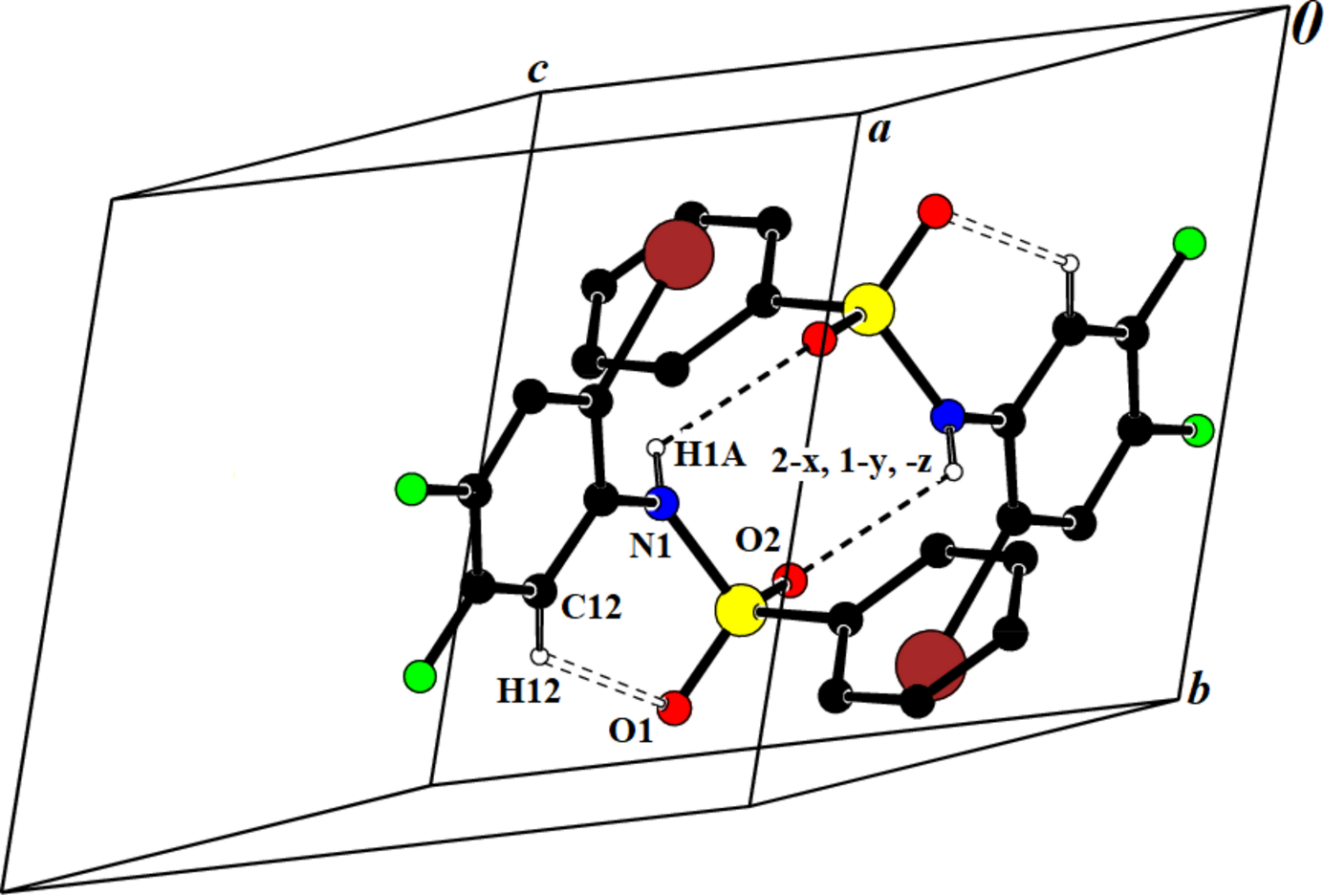
Crystal packing of compound (**II**), showing the N—H⋯O(S) hydrogen-bonding inter­actions that lead to inversion-related dimers.

In the crystal structure of (**II**), mol­ecules are linked by N—H⋯O(S) hydrogen-bonding inter­actions (Table 2 ▸, Fig. 4 ▸) into inversion-related dimers with an 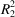
(8) graph-set motif (Etter *et al.*, 1990 ▸). Like for (**I**), π–π inter­actions are present that consolidate the crystal packing, here with centroid-to-centroid distances *Cg*1⋯*C*g1(1 − *x*, 2 − *y*, −*z*) = 3.621 (2) and a slippage of 0.998 Å and *Cg*2⋯*Cg*2(2 − *x*, 1 − *y*, 1 − *z*) = 3.797 (2) Å and a slippage of 1.617 Å (*Cg*1 and *Cg*2 are the centroids of the C1–C6 and C7–C12 rings, respectively).

## 4. Hirshfeld surface analysis

In order to qu­antify the inter­molecular inter­actions in the crystals of (**I**) and (**II**), Hirshfeld surfaces and two-dimensional fingerprint plots were generated using *CrystalExplorer* (Spackman *et al.*, 2021 ▸).

**Figure 5:**
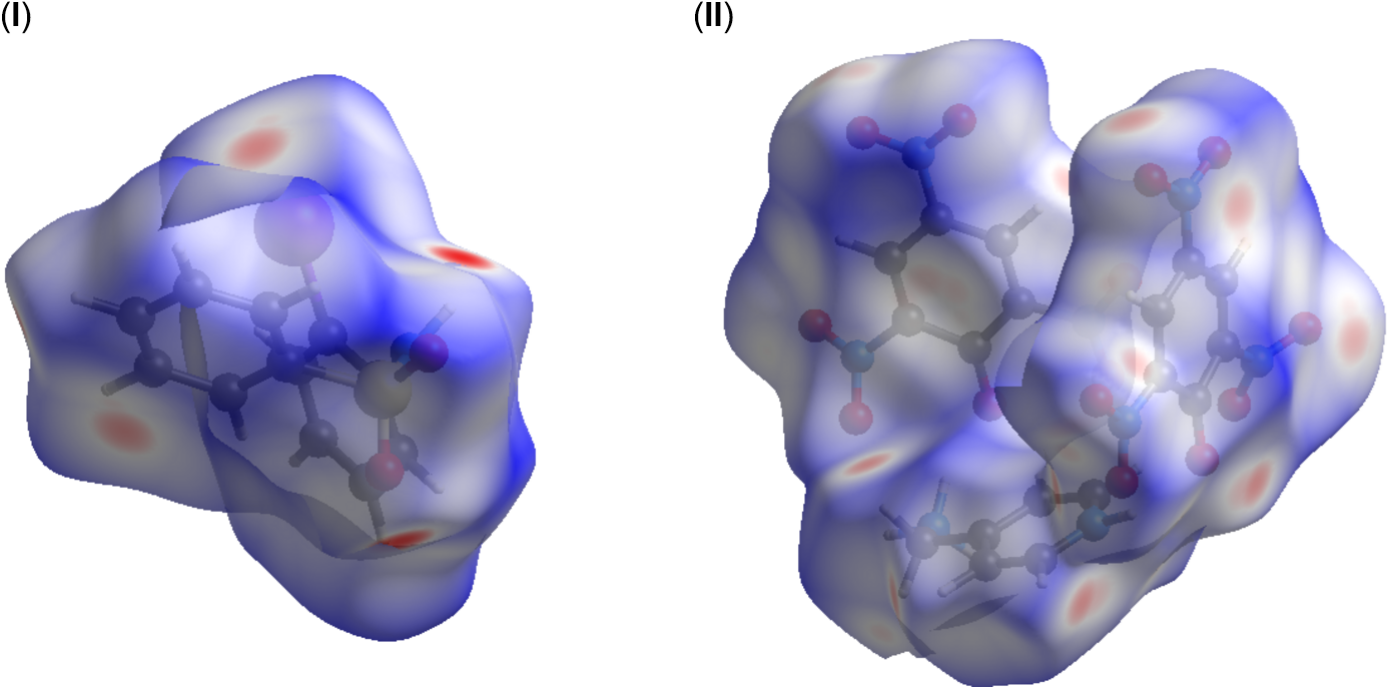
The Hirshfeld surfaces of compounds (**I**) and (**II**) mapped over *d*_norm_.

**Figure 6:**
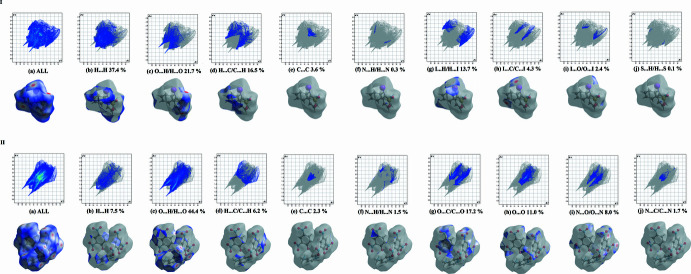
Two-dimensional fingerprint plots for (**I**) and delineated into the principal contributions of H⋯H, O⋯H/H⋯O, C⋯H/H⋯C, I⋯H/H⋯I, I⋯C/C⋯I, C⋯C and I⋯O/O⋯I contacts and for (**II**) O⋯H/H⋯O, O⋯C/C⋯O, O⋯O, N⋯O/O⋯N, H⋯H, H⋯C/C⋯H, C⋯C, N⋯C/C⋯N and N⋯H/H⋯N. Other contributors account for less than 1.0% contacts to the surface areas.

Plots of *d*_norm_ use the normalized functions *d*_i_ and *d*_e_ (Fig. 5 ▸), with white surfaces indicating contacts with distances equal to the sum of van der Waals (vdW) radii, while red and blue colors reflect contacts at the distances below and above sum of the corresponding vdW radii, respectively. Two-dimensional fingerprint plots showing the occurrence of all inter­molecular contacts (McKinnon *et al.*, 2007 ▸) and are presented in Fig. 6 ▸. H⋯H in (**I**) and O⋯H/H⋯O contacts in (**II**) represent the largest contributions to the Hirshfeld surfaces (37.4 and 44.7%, respectively). Beyond these largest fractions, short contacts are O⋯H/H⋯O (21.7%) for (**I**) and O⋯C/C⋯O (17.2%) for (**II**) (Fig. 6 ▸*c*), C⋯H/H⋯C (16.5%) for (**I**) and O⋯O (11%) for (**II**) (Fig. 6 ▸*d*). The significant increase in the O⋯H/H⋯O contributions when moving from (**I**) to (**II**) reflects growing significance of C—H⋯O binding. This is in line with a larger number of the available inter­molecular O-atom acceptors in the latter case. Accordingly, a pair of spikes identifying O⋯H/H⋯O contacts on the plots in the case of (**I)** is more diffuse.

In brief, the Hirshfeld surface analyses complement the main merit of the structure analyses, and together they suggest possibilities for controlling the supra­molecular behavior of benzene­sulfonamide as possible biomedical materials.

## 5. Database survey

A search of the Cambridge Structural Database (CSD, version 5.37; Groom *et al.*, 2016 ▸) indicated 123 compounds incorporating the phenyl­sulfonamide moiety. The bond lengths and angles in (**I**) and (**II**) are very close to those observed in 2,4-dimethyl-*N*-(phen­yl)benzene­sulfonamide (Gowda *et al.*, 2009*a* ▸), 4-chloro-2-methyl-*N*-(phen­yl)benzene­sulfonamide (Gowda *et al.*, 2009*b* ▸), 4-methyl-*N*-(3,4-di­methyl­phen­yl)benzene­sulfonamide (Gowda *et al.*, 2009*c* ▸) and other aryl sulfonamides Perlovich *et al.*, 2006 ▸; Tatsuta *et al.*, 2009 ▸; Arora & Sundaralingam, 1971 ▸; Gelbrich *et al.*, 2007 ▸). Of these, the most closely related examples are provided by structures of bromo­substituted 3-methyl-1-(phenyl­sulfon­yl)-1*H*-indole derivatives (Madhan *et al.*, 2024*b* ▸).

## 6. Synthesis and crystallization

(**I**): To a solution of 2-iodo­aniline (2 g, 9.17 mmol) in dry di­chloro­methane (DCM; 10 ml), benzene­sulfonyl chloride (1.42 ml, 11.01 mmol) and pyridine (1.11 ml, 13.76 mmol) were slowly added and stirred at room temperature for 8 h under nitro­gen atmosphere. After completion of the reaction (monitored by TLC), it was poured into ice water containing conc. HCl (1 ml), extracted with DCM (3 × 10 ml) then washed with water (2 × 20 ml) and dried (Na_2_SO_4_). Removal of the solvent *in vacuo* followed by trituration of the crude product with diethyl ether (5 ml) afforded (**I**) (2.43 g, 84%) as a colorless solid. M.p: 363–365 K. ^1^H-NMR (300 MHZ, CDCl_3_): δ 7.70 (*d*, *J* = 7.5 Hz, 2H), 7.61 (*t*, *J* = 7.5 Hz, 2H), 7.55–7.49 (*m*, 1 H), 7.41–7.36 (*m*, 2H), 7.28 (*t*, *J* = 7.5 Hz, 1H), 6.82–6.77 (*m*, 2H) ppm; 13C{1H}-NMR (75 MHz, CDCl_3_): δ 139.1, 138.8, 137.4, 133.3, 129.6, 127.4, 122.9, 92.5 ppm.

(**I**I): To a solution of 4,5-di­fluoro2-iodo­aniline (2 g, 7.84 mmol) in dry DCM (10 ml), benzene­sulfonyl chloride (1.21 ml, 9.41 mmol) and pyridine (0.95 ml, 11.76 mmol) were slowly added and stirred at room temperature for 8 h under nitro­gen atmosphere. After completion of the reaction (monitored by TLC), it was poured into ice water containing conc. HCl (1 ml), extracted with DCM (3 × 10 ml) then washed with water (2 × 20 ml) and dried (Na_2_SO_4_). Removal of solvent *in vacuo* followed by trituration of the crude product with diethyl ether (5 mL) afforded benzene­sulfonamide (**II**) (2.56 g, 83%) as a colorless solid. M.p: 393–395 K. ^1^H-NMR (300 MHZ, CDCl_3_): δ 7.58 (*d*, *J* = 7.5 Hz, 2H), 7.45–7.39 (*m*, 2 H), 7.30–7.26 (*m*, 3 H)ppm; 13C{1H}-NMR (75 MHz, CDCl_3_): δ 150.7 (*dd*, ^1^*J*_C–F_ = 249.4 Hz, ^2^*J*_C–F_ = 12.7 Hz), 147.8 (*dd*, ^1^*J*_C–F_ = 252 Hz, ^2^*J*_C–F_ = 12.7 Hz), 138.4, 134.3 (*dd*, ^1^*J*_C–F_ = 82 Hz, ^2^*J*_C–F_ = 3 Hz), 133.7, 129.3, 127.4, 126.8 (*d*, *J*_C–F_ = 19.5 Hz), 112.3 (*d*, *J*_C–F_ = 21.7 Hz) ppm.

## 7. Refinement

Crystal data, data collection and structure refinement details are summarized in Table 3 ▸. All hydrogen atoms were positioned geometrically and refined as riding with N—H = 0.86 and C—H = 0.93 Å (aromatic CH) with *U*_iso_(H) = 1.5*U*_eq_(C) for methyl groups and 1.2*U*_eq_(C) for other H atoms.

## Supplementary Material

Crystal structure: contains datablock(s) global, I, II. DOI: 10.1107/S2056989025006656/wm5762sup1.cif

Structure factors: contains datablock(s) I. DOI: 10.1107/S2056989025006656/wm5762Isup2.hkl

Structure factors: contains datablock(s) II. DOI: 10.1107/S2056989025006656/wm5762IIsup3.hkl

Supporting information file. DOI: 10.1107/S2056989025006656/wm5762Isup4.cml

Supporting information file. DOI: 10.1107/S2056989025006656/wm5762IIsup5.cml

CCDC references: 2475707, 2475706

Additional supporting information:  crystallographic information; 3D view; checkCIF report

## Figures and Tables

**Figure 2:**
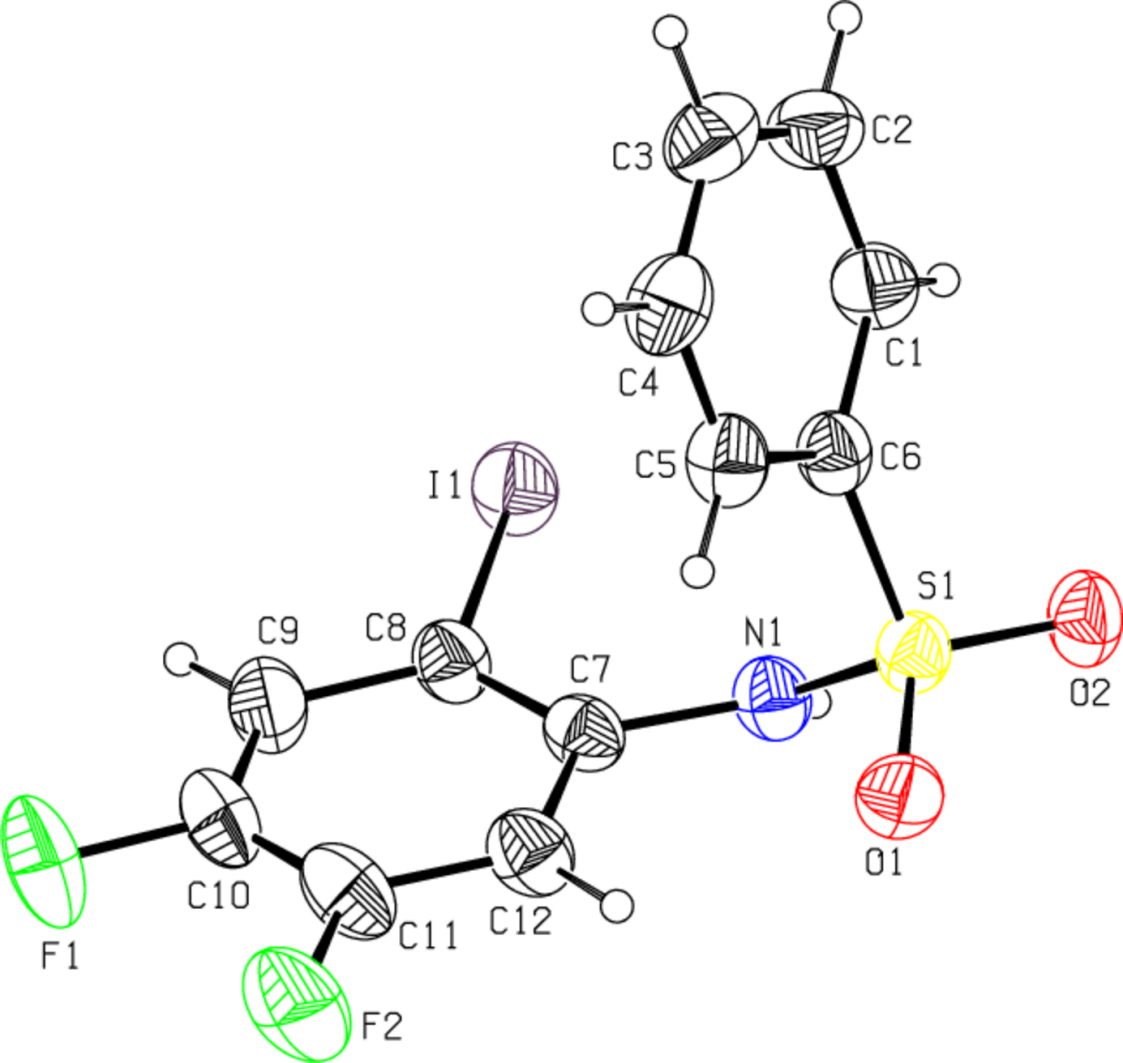
The mol­ecular structure of compound (**II**), with atom labeling and displacement ellipsoids drawn at the 50% probability level.

**Table 1:** Hydrogen-bond geometry (Å, °) for (**I**)

*D*—H⋯*A*	*D*—H	H⋯*A*	*D*⋯*A*	*D*—H⋯*A*
N1—H1*A*⋯O2^i^	0.86	2.37	3.144 (3)	149
C3—H3⋯O1^ii^	0.93	2.57	3.352 (4)	142

Symmetry codes: (i) 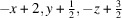; (ii) 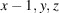.

**Table 2:** Hydrogen-bond geometry (Å, °) for (**II**)

*D*—H⋯*A*	*D*—H	H⋯*A*	*D*⋯*A*	*D*—H⋯*A*
N1—H1*A*⋯O2^i^	0.86	2.58	3.169 (8)	126
C12—H12⋯O1	0.93	2.27	2.916 (11)	126

Symmetry code: (i) 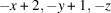.

**Table 3:** Experimental details

	(**I**)	(**II**)
Crystal data		
Chemical formula	C_12_H_10_INO_2_S	C_12_H_8_F_2_INO_2_S
*M*_r_	359.17	395.15
Crystal system, space group	Monoclinic, *P*2_1_/*c*	Triclinic, *P* 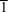
Temperature (K)	298	298
*a*, *b*, *c* (Å)	8.3648 (4), 9.8537 (4), 15.3923 (8)	8.2688 (5), 8.5178 (5), 10.4329 (7)
α, β, γ (°)	90, 90.955 (2), 90	81.291 (2), 80.503 (2), 68.383 (2)
*V* (Å^3^)	1268.52 (10)	670.47 (7)
*Z*	4	2
Radiation type	Mo *K*α	Cu *K*α
μ (mm^−1^)	2.68	20.44
Crystal size (mm)	0.35 × 0.25 × 0.08	0.22 × 0.10 × 0.05

Data collection		
Diffractometer	Bruker D8 Venture Diffractometer	Bruker D8 Venture Diffractometer
Absorption correction	Multi-scan (*SADABS*; Krause *et al.*, 2015 ▸)	Multi-scan (*SADABS*; Krause *et al.*, 2015 ▸)
*T*_min_, *T*_max_	0.672, 0.971	0.240, 0.521
No. of measured, independent and observed [*I* > 2σ(*I*)] reflections	29284, 2591, 2386	14553, 2476, 2367
*R*_int_	0.049	0.062
(sin θ/λ)_max_ (Å^−1^)	0.625	0.605

Refinement		
*R*[*F*^2^ > 2σ(*F*^2^)], *wR*(*F*^2^), *S*	0.027, 0.069, 1.09	0.072, 0.203, 1.14
No. of reflections	2591	2476
No. of parameters	155	173
H-atom treatment	H-atom parameters constrained	H-atom parameters constrained
Δρ_max_, Δρ_min_ (e Å^−3^)	0.54, −0.56	1.66, −0.95

Computer programs: *APEX2* and *SAINT* (Bruker, 2016 ▸), *SHELXT* (Sheldrick, 2015*a* ▸), *SHELXL* (Sheldrick, 2015*b* ▸), *ORTEP-3 for Windows* and *WinGX* (Farrugia, 2012 ▸), *Mercury* (Macrae *et al.*, 2020 ▸), *publCIF* (Westrip, 2010 ▸) and *PLATON* (Spek, 2009 ▸).

## supplementary crystallographic information

### N-(2-Iodophenyl)benzenesulfonamide (I) Crystal data

**Table:**

C_12_H_10_INO_2_S	*F*(000) = 696
*M**_r_* = 359.17	*D*_x_ = 1.881 Mg m^−^^3^
Monoclinic, *P*2_1_/*c*	Mo *K*α radiation, λ = 0.71073 Å
*a* = 8.3648 (4) Å	Cell parameters from 29284 reflections
*b* = 9.8537 (4) Å	θ = 1.4–25.0°
*c* = 15.3923 (8) Å	µ = 2.68 mm^−^^1^
β = 90.955 (2)°	*T* = 298 K
*V* = 1268.52 (10) Å^3^	Prism, colourless
*Z* = 4	0.35 × 0.25 × 0.08 mm

### N-(2-Iodophenyl)benzenesulfonamide (I) Data collection

**Table:**

Bruker D8 Venture Diffractometer	2386 reflections with *I* > 2σ(*I*)
Radiation source: micro focus sealed tube	*R*_int_ = 0.049
ω and φ scans	θ_max_ = 26.4°, θ_min_ = 3.4°
Absorption correction: multi-scan (*SADABS*; Krause *et al.*, 2015)	*h* = −10→10
*T*_min_ = 0.672, *T*_max_ = 0.971	*k* = −12→12
29284 measured reflections	*l* = −19→19
2591 independent reflections	

### N-(2-Iodophenyl)benzenesulfonamide (I) Refinement

**Table:**

Refinement on *F*^2^	Hydrogen site location: inferred from neighbouring sites
Least-squares matrix: full	H-atom parameters constrained
*R*[*F*^2^ > 2σ(*F*^2^)] = 0.027	*w* = 1/[σ^2^(*F*_o_^2^) + (0.0319*P*)^2^ + 1.2298*P*] where *P* = (*F*_o_^2^ + 2*F*_c_^2^)/3
*wR*(*F*^2^) = 0.069	(Δ/σ)_max_ = 0.002
*S* = 1.09	Δρ_max_ = 0.54 e Å^−^^3^
2591 reflections	Δρ_min_ = −0.56 e Å^−^^3^
155 parameters	Extinction correction: SHELXL-2019/2 (Sheldrick 2015b), Fc^*^=kFc[1+0.001xFc^2^λ^3^/sin(2θ)]^-1/4^
0 restraints	Extinction coefficient: 0.0085 (7)

### N-(2-Iodophenyl)benzenesulfonamide (I) Special details

**Table:**

Geometry. All esds (except the esd in the dihedral angle between two l.s. planes) are estimated using the full covariance matrix. The cell esds are taken into account individually in the estimation of esds in distances, angles and torsion angles; correlations between esds in cell parameters are only used when they are defined by crystal symmetry. An approximate (isotropic) treatment of cell esds is used for estimating esds involving l.s. planes.

### N-(2-Iodophenyl)benzenesulfonamide (I) Fractional atomic coordinates and isotropic or equivalent isotropic displacement parameters (Å^2^)

**Table:**

	*x*	*y*	*z*	*U*_iso_*/*U*_eq_	
C1	0.5786 (3)	0.4285 (3)	0.74857 (19)	0.0393 (6)	
H1	0.601455	0.365298	0.705621	0.047*	
C2	0.4228 (4)	0.4717 (4)	0.7611 (2)	0.0488 (8)	
H2	0.340905	0.439224	0.725207	0.059*	
C3	0.3886 (4)	0.5624 (4)	0.8263 (3)	0.0539 (9)	
H3	0.283767	0.590431	0.834558	0.065*	
C4	0.5092 (4)	0.6113 (4)	0.8791 (3)	0.0576 (9)	
H4	0.485310	0.671374	0.923622	0.069*	
C5	0.6662 (4)	0.5720 (3)	0.8666 (2)	0.0451 (7)	
H5	0.747984	0.606424	0.901827	0.054*	
C6	0.6996 (3)	0.4810 (3)	0.80115 (17)	0.0310 (5)	
C7	0.8849 (3)	0.4506 (3)	0.61007 (17)	0.0311 (5)	
C8	0.7791 (3)	0.5277 (3)	0.55998 (18)	0.0342 (6)	
C9	0.7163 (4)	0.4759 (4)	0.4824 (2)	0.0481 (8)	
H9	0.646575	0.528401	0.448826	0.058*	
C10	0.7567 (4)	0.3479 (4)	0.4552 (2)	0.0547 (9)	
H10	0.713859	0.313449	0.403539	0.066*	
C11	0.8612 (5)	0.2705 (4)	0.5049 (2)	0.0537 (9)	
H11	0.888068	0.183527	0.486772	0.064*	
C12	0.9263 (4)	0.3215 (3)	0.5814 (2)	0.0420 (7)	
H12	0.998086	0.269303	0.613894	0.050*	
N1	0.9533 (3)	0.4996 (2)	0.69018 (14)	0.0316 (5)	
H1A	1.022622	0.564040	0.689506	0.038*	
O1	0.9978 (2)	0.4978 (2)	0.84793 (13)	0.0405 (5)	
O2	0.9031 (3)	0.28915 (19)	0.77277 (14)	0.0408 (5)	
S1	0.90001 (7)	0.43340 (6)	0.78303 (4)	0.02932 (16)	
I1	0.71045 (2)	0.72190 (2)	0.59939 (2)	0.04598 (11)	

### N-(2-Iodophenyl)benzenesulfonamide (I) Atomic displacement parameters (Å^2^)

**Table:**

	*U*^11^	*U*^22^	*U*^33^	*U*^12^	*U*^13^	*U*^23^
C1	0.0361 (15)	0.0437 (16)	0.0381 (15)	−0.0119 (12)	0.0004 (12)	0.0013 (13)
C2	0.0328 (15)	0.059 (2)	0.0542 (19)	−0.0143 (14)	−0.0079 (13)	0.0148 (16)
C3	0.0307 (15)	0.055 (2)	0.076 (2)	0.0046 (14)	0.0086 (15)	0.0110 (18)
C4	0.0435 (18)	0.057 (2)	0.072 (2)	0.0047 (16)	0.0105 (17)	−0.0175 (19)
C5	0.0360 (15)	0.0491 (18)	0.0500 (17)	−0.0010 (13)	0.0013 (13)	−0.0111 (15)
C6	0.0283 (12)	0.0328 (13)	0.0320 (13)	−0.0013 (10)	0.0003 (10)	0.0059 (11)
C7	0.0291 (12)	0.0344 (14)	0.0300 (13)	−0.0044 (10)	0.0080 (10)	−0.0005 (11)
C8	0.0319 (13)	0.0387 (15)	0.0320 (13)	−0.0018 (11)	0.0063 (11)	0.0002 (11)
C9	0.0418 (16)	0.068 (2)	0.0343 (15)	−0.0020 (15)	−0.0015 (12)	−0.0053 (15)
C10	0.053 (2)	0.069 (2)	0.0419 (17)	−0.0123 (18)	0.0080 (15)	−0.0218 (17)
C11	0.060 (2)	0.0462 (19)	0.056 (2)	−0.0063 (15)	0.0219 (17)	−0.0208 (16)
C12	0.0412 (16)	0.0392 (15)	0.0461 (16)	0.0024 (13)	0.0133 (13)	−0.0029 (13)
N1	0.0285 (11)	0.0322 (11)	0.0340 (11)	−0.0050 (9)	0.0015 (9)	0.0027 (9)
O1	0.0329 (10)	0.0483 (12)	0.0399 (11)	−0.0002 (9)	−0.0090 (8)	0.0000 (9)
O2	0.0485 (12)	0.0293 (10)	0.0445 (11)	0.0047 (8)	−0.0005 (9)	0.0073 (8)
S1	0.0274 (3)	0.0295 (3)	0.0309 (3)	0.0011 (2)	−0.0029 (2)	0.0029 (3)
I1	0.04805 (15)	0.03807 (15)	0.05150 (16)	0.00919 (8)	−0.00858 (9)	0.00039 (8)

### N-(2-Iodophenyl)benzenesulfonamide (I) Geometric parameters (Å, º)

**Table:**

C1—C6	1.385 (4)	C7—N1	1.434 (3)
C1—C2	1.388 (5)	C8—C9	1.393 (4)
C1—H1	0.9300	C8—I1	2.091 (3)
C2—C3	1.377 (5)	C9—C10	1.373 (5)
C2—H2	0.9300	C9—H9	0.9300
C3—C4	1.372 (5)	C10—C11	1.382 (6)
C3—H3	0.9300	C10—H10	0.9300
C4—C5	1.386 (5)	C11—C12	1.384 (5)
C4—H4	0.9300	C11—H11	0.9300
C5—C6	1.380 (4)	C12—H12	0.9300
C5—H5	0.9300	N1—S1	1.640 (2)
C6—S1	1.768 (3)	N1—H1A	0.8600
C7—C8	1.390 (4)	O1—S1	1.429 (2)
C7—C12	1.392 (4)	O2—S1	1.430 (2)
			
C6—C1—C2	118.8 (3)	C9—C8—I1	118.9 (2)
C6—C1—H1	120.6	C10—C9—C8	120.5 (3)
C2—C1—H1	120.6	C10—C9—H9	119.8
C3—C2—C1	120.5 (3)	C8—C9—H9	119.8
C3—C2—H2	119.7	C9—C10—C11	119.6 (3)
C1—C2—H2	119.7	C9—C10—H10	120.2
C4—C3—C2	120.0 (3)	C11—C10—H10	120.2
C4—C3—H3	120.0	C10—C11—C12	120.5 (3)
C2—C3—H3	120.0	C10—C11—H11	119.8
C3—C4—C5	120.6 (3)	C12—C11—H11	119.8
C3—C4—H4	119.7	C11—C12—C7	120.3 (3)
C5—C4—H4	119.7	C11—C12—H12	119.8
C6—C5—C4	119.1 (3)	C7—C12—H12	119.8
C6—C5—H5	120.5	C7—N1—S1	120.34 (18)
C4—C5—H5	120.5	C7—N1—H1A	119.8
C5—C6—C1	121.0 (3)	S1—N1—H1A	119.8
C5—C6—S1	119.4 (2)	O1—S1—O2	120.50 (13)
C1—C6—S1	119.6 (2)	O1—S1—N1	105.77 (12)
C8—C7—C12	118.9 (3)	O2—S1—N1	107.07 (13)
C8—C7—N1	122.3 (2)	O1—S1—C6	107.76 (13)
C12—C7—N1	118.8 (3)	O2—S1—C6	107.45 (13)
C7—C8—C9	120.2 (3)	N1—S1—C6	107.72 (12)
C7—C8—I1	121.0 (2)		
			
C6—C1—C2—C3	−1.8 (5)	C9—C10—C11—C12	−0.5 (5)
C1—C2—C3—C4	0.4 (5)	C10—C11—C12—C7	1.2 (5)
C2—C3—C4—C5	1.0 (6)	C8—C7—C12—C11	−0.8 (4)
C3—C4—C5—C6	−1.1 (6)	N1—C7—C12—C11	179.3 (3)
C4—C5—C6—C1	−0.4 (5)	C8—C7—N1—S1	110.1 (3)
C4—C5—C6—S1	178.5 (3)	C12—C7—N1—S1	−70.0 (3)
C2—C1—C6—C5	1.8 (4)	C7—N1—S1—O1	176.0 (2)
C2—C1—C6—S1	−177.1 (2)	C7—N1—S1—O2	46.3 (2)
C12—C7—C8—C9	−0.1 (4)	C7—N1—S1—C6	−69.0 (2)
N1—C7—C8—C9	179.7 (3)	C5—C6—S1—O1	3.3 (3)
C12—C7—C8—I1	179.6 (2)	C1—C6—S1—O1	−177.9 (2)
N1—C7—C8—I1	−0.6 (3)	C5—C6—S1—O2	134.5 (2)
C7—C8—C9—C10	0.8 (5)	C1—C6—S1—O2	−46.6 (3)
I1—C8—C9—C10	−178.9 (3)	C5—C6—S1—N1	−110.4 (2)
C8—C9—C10—C11	−0.5 (5)	C1—C6—S1—N1	68.4 (2)

### N-(2-Iodophenyl)benzenesulfonamide (I) Hydrogen-bond geometry (Å, º)

**Table:**

*D*—H···*A*	*D*—H	H···*A*	*D*···*A*	*D*—H···*A*
N1—H1*A*···O2^i^	0.86	2.37	3.144 (3)	149
C3—H3···O1^ii^	0.93	2.57	3.352 (4)	142

Symmetry codes: (i) −*x*+2, *y*+1/2, −*z*+3/2; (ii) *x*−1, *y*, *z*.

### N-(4,5-Difluoro-2-iodophenyl)benzenesulfonamide (II) Crystal data

**Table:**

C_12_H_8_F_2_INO_2_S	*Z* = 2
*M**_r_* = 395.15	*F*(000) = 380
Triclinic, *P*1	*D*_x_ = 1.957 Mg m^−^^3^
*a* = 8.2688 (5) Å	Cu *K*α radiation, λ = 1.54178 Å
*b* = 8.5178 (5) Å	Cell parameters from 14553 reflections
*c* = 10.4329 (7) Å	θ = 1.4–25.0°
α = 81.291 (2)°	µ = 20.44 mm^−^^1^
β = 80.503 (2)°	*T* = 298 K
γ = 68.383 (2)°	Prism, colourless
*V* = 670.47 (7) Å^3^	0.22 × 0.10 × 0.05 mm

### N-(4,5-Difluoro-2-iodophenyl)benzenesulfonamide (II) Data collection

**Table:**

Bruker D8 Venture Diffractometer	2367 reflections with *I* > 2σ(*I*)
Radiation source: micro focus sealed tube	*R*_int_ = 0.062
ω and φ scans	θ_max_ = 69.0°, θ_min_ = 4.3°
Absorption correction: multi-scan (*SADABS*; Krause *et al.*, 2015)	*h* = −9→10
*T*_min_ = 0.240, *T*_max_ = 0.521	*k* = −10→10
14553 measured reflections	*l* = −12→12
2476 independent reflections	

### N-(4,5-Difluoro-2-iodophenyl)benzenesulfonamide (II) Refinement

**Table:**

Refinement on *F*^2^	Hydrogen site location: inferred from neighbouring sites
Least-squares matrix: full	H-atom parameters constrained
*R*[*F*^2^ > 2σ(*F*^2^)] = 0.072	*w* = 1/[σ^2^(*F*_o_^2^) + (0.1461*P*)^2^ + 0.5316*P*] where *P* = (*F*_o_^2^ + 2*F*_c_^2^)/3
*wR*(*F*^2^) = 0.203	(Δ/σ)_max_ < 0.001
*S* = 1.14	Δρ_max_ = 1.66 e Å^−^^3^
2476 reflections	Δρ_min_ = −0.94 e Å^−^^3^
173 parameters	Extinction correction: SHELXL-2019/2 (Sheldrick 2015b), Fc^*^=kFc[1+0.001xFc^2^λ^3^/sin(2θ)]^-1/4^
0 restraints	Extinction coefficient: 0.017 (2)

### N-(4,5-Difluoro-2-iodophenyl)benzenesulfonamide (II) Special details

**Table:**

Geometry. All esds (except the esd in the dihedral angle between two l.s. planes) are estimated using the full covariance matrix. The cell esds are taken into account individually in the estimation of esds in distances, angles and torsion angles; correlations between esds in cell parameters are only used when they are defined by crystal symmetry. An approximate (isotropic) treatment of cell esds is used for estimating esds involving l.s. planes.

### N-(4,5-Difluoro-2-iodophenyl)benzenesulfonamide (II) Fractional atomic coordinates and isotropic or equivalent isotropic displacement parameters (Å^2^)

**Table:**

	*x*	*y*	*z*	*U*_iso_*/*U*_eq_	
C1	0.6043 (11)	0.6893 (10)	0.0209 (8)	0.0690 (17)	
H1	0.692052	0.606880	−0.025462	0.083*	
C2	0.4337 (12)	0.7289 (12)	0.0029 (10)	0.079 (2)	
H2	0.405262	0.675110	−0.056593	0.095*	
C3	0.3033 (11)	0.8488 (11)	0.0732 (10)	0.081 (2)	
H3	0.186655	0.875898	0.061633	0.097*	
C4	0.3467 (10)	0.9284 (11)	0.1607 (10)	0.080 (2)	
H4	0.257809	1.008690	0.208075	0.096*	
C5	0.5138 (10)	0.8932 (9)	0.1794 (8)	0.0674 (16)	
H5	0.540488	0.948502	0.238810	0.081*	
C6	0.6479 (9)	0.7712 (8)	0.1076 (6)	0.0582 (14)	
C7	0.8376 (8)	0.5366 (8)	0.3596 (6)	0.0571 (14)	
C8	0.7924 (10)	0.3964 (9)	0.4144 (7)	0.0614 (15)	
C9	0.7196 (12)	0.3840 (12)	0.5445 (8)	0.078 (2)	
H9	0.692000	0.289102	0.581056	0.093*	
C10	0.6899 (13)	0.5163 (13)	0.6173 (8)	0.081 (2)	
C11	0.7353 (14)	0.6529 (12)	0.5661 (9)	0.082 (2)	
C12	0.8116 (11)	0.6642 (10)	0.4384 (8)	0.0700 (18)	
H12	0.845408	0.756575	0.405198	0.084*	
N1	0.9216 (7)	0.5461 (7)	0.2298 (6)	0.0597 (12)	
H1A	1.004065	0.457002	0.202938	0.072*	
O1	0.8802 (7)	0.8532 (7)	0.1911 (6)	0.0691 (12)	
O2	0.9746 (7)	0.6771 (7)	0.0082 (5)	0.0717 (13)	
S1	0.8673 (2)	0.7215 (2)	0.12842 (16)	0.0582 (5)	
I1	0.83499 (6)	0.19041 (6)	0.30905 (4)	0.0749 (4)	
F1	0.6177 (10)	0.5090 (9)	0.7433 (5)	0.116 (2)	
F2	0.7077 (11)	0.7780 (9)	0.6410 (6)	0.112 (2)	

### N-(4,5-Difluoro-2-iodophenyl)benzenesulfonamide (II) Atomic displacement parameters (Å^2^)

**Table:**

	*U*^11^	*U*^22^	*U*^33^	*U*^12^	*U*^13^	*U*^23^
C1	0.085 (5)	0.061 (4)	0.068 (4)	−0.032 (3)	−0.006 (3)	−0.014 (3)
C2	0.089 (5)	0.080 (5)	0.083 (5)	−0.043 (4)	−0.024 (4)	−0.005 (4)
C3	0.064 (4)	0.076 (5)	0.102 (6)	−0.028 (4)	−0.017 (4)	0.007 (4)
C4	0.061 (4)	0.074 (5)	0.091 (6)	−0.016 (3)	0.008 (4)	−0.010 (4)
C5	0.074 (4)	0.057 (3)	0.068 (4)	−0.020 (3)	0.004 (3)	−0.014 (3)
C6	0.067 (3)	0.054 (3)	0.054 (3)	−0.023 (3)	−0.001 (3)	−0.007 (3)
C7	0.056 (3)	0.059 (3)	0.053 (3)	−0.012 (2)	−0.007 (2)	−0.015 (3)
C8	0.070 (4)	0.061 (4)	0.053 (3)	−0.022 (3)	−0.004 (3)	−0.010 (3)
C9	0.092 (5)	0.084 (5)	0.059 (4)	−0.038 (4)	0.002 (4)	−0.006 (4)
C10	0.095 (5)	0.092 (6)	0.048 (4)	−0.026 (4)	0.001 (3)	−0.014 (4)
C11	0.100 (6)	0.078 (5)	0.060 (4)	−0.015 (4)	−0.011 (4)	−0.023 (4)
C12	0.085 (5)	0.062 (4)	0.063 (4)	−0.024 (3)	−0.008 (3)	−0.014 (3)
N1	0.065 (3)	0.056 (3)	0.055 (3)	−0.018 (2)	0.000 (2)	−0.014 (2)
O1	0.079 (3)	0.064 (3)	0.073 (3)	−0.033 (2)	−0.002 (2)	−0.020 (2)
O2	0.078 (3)	0.076 (3)	0.059 (3)	−0.030 (3)	0.014 (2)	−0.018 (2)
S1	0.0632 (9)	0.0577 (9)	0.0560 (9)	−0.0254 (7)	0.0028 (7)	−0.0127 (7)
I1	0.0903 (5)	0.0698 (5)	0.0695 (5)	−0.0344 (3)	0.0010 (3)	−0.0166 (3)
F1	0.154 (6)	0.122 (5)	0.052 (3)	−0.036 (4)	0.022 (3)	−0.018 (3)
F2	0.156 (6)	0.104 (4)	0.077 (3)	−0.038 (4)	−0.001 (3)	−0.045 (3)

### N-(4,5-Difluoro-2-iodophenyl)benzenesulfonamide (II) Geometric parameters (Å, º)

**Table:**

C1—C2	1.363 (12)	C7—N1	1.422 (9)
C1—C6	1.386 (10)	C8—C9	1.396 (10)
C1—H1	0.9300	C8—I1	2.100 (7)
C2—C3	1.377 (14)	C9—C10	1.378 (13)
C2—H2	0.9300	C9—H9	0.9300
C3—C4	1.376 (14)	C10—F1	1.353 (9)
C3—H3	0.9300	C10—C11	1.360 (15)
C4—C5	1.343 (12)	C11—F2	1.346 (10)
C4—H4	0.9300	C11—C12	1.382 (12)
C5—C6	1.408 (10)	C12—H12	0.9300
C5—H5	0.9300	N1—S1	1.655 (6)
C6—S1	1.748 (7)	N1—H1A	0.8600
C7—C12	1.394 (10)	O1—S1	1.425 (5)
C7—C8	1.394 (10)	O2—S1	1.425 (5)
			
C2—C1—C6	120.4 (8)	C9—C8—I1	116.3 (6)
C2—C1—H1	119.8	C10—C9—C8	118.2 (8)
C6—C1—H1	119.8	C10—C9—H9	120.9
C1—C2—C3	119.9 (8)	C8—C9—H9	120.9
C1—C2—H2	120.1	F1—C10—C11	118.9 (9)
C3—C2—H2	120.1	F1—C10—C9	119.6 (9)
C4—C3—C2	119.7 (7)	C11—C10—C9	121.4 (8)
C4—C3—H3	120.2	F2—C11—C10	119.7 (8)
C2—C3—H3	120.2	F2—C11—C12	119.5 (9)
C5—C4—C3	121.8 (8)	C10—C11—C12	120.8 (8)
C5—C4—H4	119.1	C11—C12—C7	119.7 (8)
C3—C4—H4	119.1	C11—C12—H12	120.1
C4—C5—C6	118.9 (8)	C7—C12—H12	120.1
C4—C5—H5	120.5	C7—N1—S1	122.7 (4)
C6—C5—H5	120.5	C7—N1—H1A	118.7
C1—C6—C5	119.3 (7)	S1—N1—H1A	118.7
C1—C6—S1	120.1 (6)	O1—S1—O2	118.7 (4)
C5—C6—S1	120.6 (5)	O1—S1—N1	107.5 (3)
C12—C7—C8	118.5 (7)	O2—S1—N1	105.5 (3)
C12—C7—N1	119.4 (7)	O1—S1—C6	108.4 (3)
C8—C7—N1	121.8 (6)	O2—S1—C6	109.5 (3)
C7—C8—C9	121.3 (7)	N1—S1—C6	106.5 (3)
C7—C8—I1	122.4 (5)		
			
C6—C1—C2—C3	−1.2 (12)	F1—C10—C11—C12	179.1 (9)
C1—C2—C3—C4	0.3 (13)	C9—C10—C11—C12	0.7 (15)
C2—C3—C4—C5	0.4 (14)	F2—C11—C12—C7	−178.8 (8)
C3—C4—C5—C6	−0.2 (13)	C10—C11—C12—C7	2.0 (13)
C2—C1—C6—C5	1.4 (11)	C8—C7—C12—C11	−2.8 (11)
C2—C1—C6—S1	−179.2 (6)	N1—C7—C12—C11	−177.8 (7)
C4—C5—C6—C1	−0.7 (11)	C12—C7—N1—S1	−47.0 (8)
C4—C5—C6—S1	179.9 (6)	C8—C7—N1—S1	138.2 (6)
C12—C7—C8—C9	1.2 (11)	C7—N1—S1—O1	55.0 (6)
N1—C7—C8—C9	176.1 (7)	C7—N1—S1—O2	−177.4 (5)
C12—C7—C8—I1	−177.3 (5)	C7—N1—S1—C6	−61.1 (6)
N1—C7—C8—I1	−2.4 (9)	C1—C6—S1—O1	163.4 (6)
C7—C8—C9—C10	1.3 (13)	C5—C6—S1—O1	−17.1 (7)
I1—C8—C9—C10	179.9 (7)	C1—C6—S1—O2	32.5 (7)
C8—C9—C10—F1	179.3 (8)	C5—C6—S1—O2	−148.0 (6)
C8—C9—C10—C11	−2.3 (14)	C1—C6—S1—N1	−81.1 (6)
F1—C10—C11—F2	−0.1 (15)	C5—C6—S1—N1	98.4 (6)
C9—C10—C11—F2	−178.6 (9)		

### N-(4,5-Difluoro-2-iodophenyl)benzenesulfonamide (II) Hydrogen-bond geometry (Å, º)

**Table:**

*D*—H···*A*	*D*—H	H···*A*	*D*···*A*	*D*—H···*A*
N1—H1*A*···O2^i^	0.86	2.58	3.169 (8)	126
C12—H12···O1	0.93	2.27	2.916 (11)	126

Symmetry code: (i) −*x*+2, −*y*+1, −*z*.

### Geometry of stacking interactions for I and II (Å, °).

**Table:**

Compound	Group A	Group B	Shortest contacts	CgA···CgB	Plane···CgB	ipa	sa
I	(C7–C12)	(C7–C12)^ii^	3.747 (2)	3.747 (2)	3.602 (2)	0	16.0 (1)
II	(C1–C6)	(C1–C6)^ˆiii^	3.225 (2)	3.621 (2)	3.480 (2)	0	16.0 (2)
	(C7–C12)	(C7–C12)^iv^	3.499 (2)	3.797 (2)	3.436 (2)	0	25.2 (2)

a) Cg is a group centroid; Plane···CgB is the distance between mean plane of Group A and centroid of the interacting Group B; ipa is interplanar angle; sa is slippage angle, which is the angle of CgA···CgB axis to the Group A mean plane normal. [Symmetry codes for I: (ii) 2-X,-Y,-Z; for II: (iii) 1-X,2-Y,-Z; (iv) 2-X,1-Y,1-Z.]
